# The C-Terminal Domain from *S. cerevisiae* Pat1 Displays Two Conserved Regions Involved in Decapping Factor Recruitment

**DOI:** 10.1371/journal.pone.0096828

**Published:** 2014-05-15

**Authors:** Zaineb Fourati, Olga Kolesnikova, Régis Back, Jenny Keller, Clément Charenton, Valerio Taverniti, Claudine Gaudon Plesse, Noureddine Lazar, Dominique Durand, Herman van Tilbeurgh, Bertrand Séraphin, Marc Graille

**Affiliations:** 1 Laboratoire de Biochimie, Centre National de Recherche Scientifique (CNRS) UMR 7654, Ecole Polytechnique, Palaiseau Cedex, France; 2 Institut de Biochimie et Biophysique Moléculaire et Cellulaire (IBBMC), Centre National de Recherche Scientifique (CNRS) UMR 8619, Bat. 430, Université Paris Sud, Orsay Cedex, France; 3 Equipe Labellisée La Ligue, Institut de Génétique et de Biologie Moléculaire et Cellulaire (IGBMC), Centre National de Recherche Scientifique (CNRS) UMR 7104/Institut National de Santé et de Recherche Médicale (INSERM) U964/Université de Strasbourg, Illkirch, France; Colorado State University, United States of America

## Abstract

Eukaryotic mRNA decay is a highly regulated process allowing cells to rapidly modulate protein production in response to internal and environmental cues. Mature translatable eukaryotic mRNAs are protected from fast and uncontrolled degradation in the cytoplasm by two cis-acting stability determinants: a methylguanosine (m^7^G) cap and a poly(A) tail at their 5′ and 3′ extremities, respectively. The hydrolysis of the m^7^G cap structure, known as decapping, is performed by the complex composed of the Dcp2 catalytic subunit and its partner Dcp1. The Dcp1-Dcp2 decapping complex has a low intrinsic activity and requires accessory factors to be fully active. Among these factors, Pat1 is considered to be a central scaffolding protein involved in Dcp2 activation but also in inhibition of translation initiation. Here, we present the structural and functional study of the C-terminal domain from *S. cerevisiae* Pat1 protein. We have identified two conserved and functionally important regions located at both extremities of the domain. The first region is involved in binding to Lsm1-7 complex. The second patch is specific for fungal proteins and is responsible for Pat1 interaction with Edc3. These observations support the plasticity of the protein interaction network involved in mRNA decay and show that evolution has extended the C-terminal alpha-helical domain from fungal Pat1 proteins to generate a new binding platform for protein partners.

## Introduction

Protein synthesis in eukaryotic cells is finely tuned both by controlling mRNA levels and their translation. The steady-state level of a given mRNA is governed by its rates of transcription and degradation. Hence, the regulation of mRNA decay provides a rapid mechanism to adapt to changing conditions. Eukaryotic mature mRNAs are protected from fast and uncontrolled degradation in the cytoplasm by two main *cis*-acting stability determinants: a methylguanosine (m^7^G) cap and a poly(A) tail present at their 5′ and 3′ extremities, respectively. Bulk mRNA decay is initiated by 3′ poly(A) tail shortening followed by degradation via either the 3′→5′ or 5′→3′ mRNA decay mechanisms [1]. In the 3′→5′ mRNA decay, the mRNA is degraded by the exosome, a multiprotein complex with both exo- and endo-nucleolytic activities embedded within a single protein Dis3 [2], [3], [4], [5], [6]. This generates a 5′ capped oligonucleotide byproduct (less than 10 base long) that is further degraded by the scavenger protein DcpS (Dcs1 in budding yeast) to release m^7^GMP [7]. In the 5′→3′ pathway, the 5′ cap is eliminated by a critical and highly regulated step known as decapping, which is followed by the rapid digestion of the mRNA body from its 5′ end, a process mediated by the exonuclease Xrn1 [8], [9].

The decapping step consists in the hydrolysis of the cap structure generating m^7^GDP and a 5′ phosphorylated RNA molecule. This reaction is performed by the decapping holoenzyme composed by the catalytic subunit Dcp2 and its co-factor Dcp1 [10], [11], [12]. This complex has a low intrinsic decapping activity and requires one or several accessory factors (such as Lsm1-7, Pat1 or Edc1-4 proteins) to be fully efficient [13], [14]. In particular, deletion of the *LSM1-7* genes results in mRNAs stabilization associated with accumulation of deadenylated capped mRNAs [15], [16]. These genes encode for Lsm proteins, which associate to form the heteroheptameric Lsm1-7 complex. This complex binds preferentially to deadenylated or oligoadenylated, but not polyadenylated, mRNAs [17], [18] and interacts with Pat1 [15], [16], [19]. Edc3 is another activator of decapping but *EDC3* deletion does not result in the general accumulation of capped mRNA decay intermediates. Edc3 contains an Sm/Lsm domain and interacts with the yeast DEAD-box helicase Dhh1 (RCK/p54/DDX6 in mammals, Me31B in fruit fly) and Pat1 (Pat1b in mammals, HPat in fruit fly) [20], [21], [22], [23]. Edc3 was also proposed to associate to Dcp2 via its Lsm domain and to stimulate thereby its enzymatic activity *in vitro* [24], [25], [26]. Finally, in fungi Edc3 was shown to regulate the *YRA1* pre-mRNA level and to interact with the ribosomal protein Rps28 participating thereby to an autoregulatory loop controlling the decapping of mRNAs encoding this protein [27], [28], [29], [30]. Additional decapping activators are specific to fungi or metazoans. In yeast, this includes the RNA-binding proteins Edc1 and Edc2 that stimulate Dcp2 activity *in vitro* [31]. In contrast, metazoan EDC4 (also known as Ge-1 or Hedls proteins) stimulates DCP2 by promoting its association with DCP1 [32], [33].

The deletion of the *PAT1* gene in *S. cerevisiae* results in a thermosensitive growth phenotype associated with a strong decapping inhibition *in vivo* [15], [16], [19]. These observations revealed that Pat1 is a critical component of the decapping machinery. As Pat1 interacts with numerous mRNA decay factors including the Lsm1-7 complex, the 5′→3′ exonuclease Xrn1, the DEAD-box helicase Dhh1, Edc3, and Dcp2 in yeast and other organisms [15], [16], [19], [21], [22], [23], [26], [34], [35], it appears to function as a scaffold protein allowing these proteins to form a multisubunit assembly that mediates regulated mRNA decapping. Pat1 is composed of several regions (domains N for N-terminal, M for Middle and C for C-terminal) that are responsible for interaction with various partners and that are implicated in distinct Pat1 functions. The interacting network formed by Pat1 from yeast, drosophila and human and its partners has been dissected using various strategies: co-immunoprecipitation of proteins expressed in insect cells, interaction analysis using recombinant *S. cerevisiae* proteins or domains, and yeast two-hybrid assays [21], [26], [34], [36], [37]. Dhh1 proteins from the three species interact with the N-terminal domain of their Pat1 counterparts and this interaction was proposed to modulate the interaction of Dhh1 with RNA [20], [22], [34], [35], [36]. Other common partners of Pat1 in these three species include the Lsm1-7 protein complex and subunits of the decapping complex (containing Dcp1, Dcp2, and in addition EDC4 in mammals; [38]). This is consistent with the requirement of Pat1 and the Lsm proteins for decapping *in vivo* [15], [16], [19] and stimulation of *in vitro* decapping by the *S. cerevisiae* Pat1 C domain [26]. Recent structural studies have described the interaction mode between yeast Pat1 C domain and the Lsm1-7 complex and shown that Pat1 interacts directly with the Lsm2 and Lsm3 proteins within the Lsm1-7 complex [39], [40]. Other Pat1 partners related to mRNA decay and translational control have been observed in one or two of these three species. This includes subunits of the CCR4-NOT complex (human, drosophila), Scd6 (yeast), Edc3 (yeast, human) and Xrn1 (yeast, human) [15], [20], [21], [26], [34], [41]. At this stage, it is unclear whether some interactions are species specific neither whether all these proteins interact directly with Pat1, especially as different assays were used (*e.g*., co-immunoprecipitation and two-hybrid assays versus interaction between purified recombinant factors). Pat1 proteins are also nucleic acid binding proteins. Indeed, P100, the *X. laevis* Pat1 ortholog, binds to single-stranded but not double-stranded DNA (RNA was not tested; [42]). In addition, yeast Pat1 and human Pat1b domain C bind to poly(U) RNA but not poly(C) or poly(A) [21], [36].

Altogether these proteins participate to an intricate network of protein-protein and protein-RNA interactions, which have been proposed to promote decapping by successive steps. A model suggests that Dhh1, Pat1 and Scd6 first repress translation and inhibit formation of the 48S initiation complex [26]. This is supported by the translational repression induced upon over-expression of Pat1 and Dhh1 [43], [44]. Second, remodeling of the mRNP particles leads to recruitment of new decapping factors and loss of eIF4E and eIF4G initiation factors, which were bound to the cap [45]. Finally, the Pat1 and Edc3 proteins as well as the Lsm1-7 complex activate the Dcp2 catalytic subunit [15], [16], [26]. However, independent experiments suggest that decapping can also occur on translated mRNAs [46]. Pat1 and its partners co-localize to cytoplasmic foci known as P-bodies and most of these proteins are important for formation of these mRNP granules [47],[48],[49],[50]. These P-bodies contain few translation initiation factors and are assumed to be dedicated to the storage of translationally repressed mRNAs as well as to the decapping and degradation of mRNAs.

In this paper, we have focused our attention on the *S. cerevisiae* Pat1 C domain (ScPat1C), which is required for decapping and involved in interaction with Lsm1-7 complex, Xrn1, Edc3, Dcp1 and Dcp2. We have determined its crystal structure and identified two functionally important regions located at both extremities of this domain that are important for the recruitment of different decapping factors.

## Materials and Methods

### Yeast strains and shuttling plasmid

Yeast strains used in this study are listed in Table S1. Multiple mutant strains were obtained by crossing and tetrad dissection following standard genetic procedures. Strains BSY1133 with the deletion of *PAT1* gene and YFW168 with deletions of *PAT1* and *DHH1* genes were kindly provided by Michèle Minet.

Shuttling plasmids for complementation analyses are listed in Table S2. Briefly, the yeast *PAT1* gene was amplified from yeast genomic DNA using oligonucleotides OBS4954 and OBS4955 (Table S3). The PCR product was cloned into XhoI/BamHI sites of pRS414 [51] giving pBS4357. Plasmids bearing *PAT1* gene with deletions or point mutations were obtained using the Quikchange strategy on the pBS4357 template. Oligonucleotides used in this study are listed in Table S3.

### Cell growth conditions

For complementation analyses, cells were grown to the logarithmic phase in synthetic complete (SC) medium lacking tryptophan complemented with 2% glucose. Cultures were diluted to an optical density of 0.1 at 600 nm (OD_600_) with sterile water. Three µL of each strain as well as three µL of three consecutive 10-fold serial dilutions were plated on SC medium lacking tryptophan. Cell growth rate was monitored at 25°C, 30°C and 37°C after 48 or 72 hours in case of the *Δdhh1/Δpat1* strain.

### Two-hybrid interaction analysis

Two-hybrid interaction analyses were done using standard procedure in two strains MAV203 (Invitrogen) and the isogenic BSY2475 lacking Edc3 [30]. The β-galactosidase activity was measured using Beta-Glo Assay system (Promega). Two hybrid plasmids were constructed by standard cloning techniques after PCR amplification of the coding sequences. The plasmid pBS4907, expressing *PAT1* gene lacking C-terminal 68 amino acids fused to Gal4AD, was obtained by the QuickChange strategy using OBS5012 and OBS5013 and pBS2374 as a template. Oligonucleotides used in this study are listed in Table S3.

### Cloning, expression and purification of Pat1C proteins

The coding sequence of Pat1 C-terminal domain (hereafter named ScPat1C encompassing residues 473 to 796) was amplified from yeast *Saccharomyces cerevisiae S288C* genomic DNA with oligonucleotides oMG18/oMG180 (see Table S3) and inserted into pET21-a vector with a sequence coding for a His_6_ tag at the 3′ end of the coding sequence, yielding plasmid pMG311.

The ScPat1C protein (both unlabeled or Se-Met labeled) was expressed in *E. coli* Rosetta (DE3) pLysS strain (Novagen) in 2YT medium supplemented with ampicillin at 100 µg/mL and chloramphenicol at 25 µg/mL. At OD_600_ = 0.8, the protein expression was induced during 20 h at 28°C by adding 50 µg/mL IPTG. Cells were harvested by centrifugation and resuspended in 30 mL of buffer A (20 mM Tris-HCl pH 7.5, 200 mM NaCl). Cell lysis was performed by sonication. The His_6_ tagged protein was purified on NiNTA column (Qiagen) followed by a heparin column (GE Healthcare) and finally on Superdex 200 16/60 size exclusion column (GE Healthcare) in buffer A.

The DNA sequences coding for the region encompassing residues 435 to 796 from *Saccharomyces cerevisiae* Pat1 mutants were amplified from the complementation plasmids pBS4436 (mutant Q706A/L713A), pBS4437 (mutant K475E/K476E), pBS4438 (mutant K531E/K534E/R538E), pBS4439 (mutant R497E) and pBS4440 (mutant Q720A/R721A/D725A/R728A) using oligonucleotides oMG119/oMG118 (see Table S3). They were sub-cloned into pET21-a with a sequence coding for a N-terminal His_6_ tag to yield expression plasmids pMG606 (mutant Q706A/L713A), pMG607 (mutant K475E/K476E), pMG608 (mutant K531E/K534E/R538E), pMG609 (mutant R497E) and pMG610 (mutant Q720A/R721A/D725A/R728A).

All ScPat1 [435-796] mutants were expressed in *E. coli* BL21(DE3) Codon^+^ (Novagen) in 1L of 2YT medium supplemented with ampicillin at 100 µg/mL and chloramphenicol at 25 µg/mL. At OD_600_ = 0.8, protein expression was induced at 20°C for 20 h by adding 50 µg/mL IPTG. Cells were harvested by centrifugation and resuspended in 40 mL of buffer B (20 mM Tris-HCl pH 8, 200 mM NaCl) supplemented with 20 mM imidazole. Cell lysis was performed by sonication.

These mutants were purified on a NiNTA column (Qiagen), and then on a Superdex 75 16/60 size exclusion column (GE Healthcare) equilibrated in buffer B.

### Cloning, expression and purification of the Lsm1-7 complex

A plasmid containing an operon made of the partial (Lsm4, Lsm7) or complete (Lsm1-3, Lsm5-6) coding sequences of yeast Lsm1-7 proteins was prepared by amplifying individually each coding sequence containing a ribosome binding site upstream of each start codon and unique restriction sites at both ends using oligonucleotides OBS6352 to OBS6355 and oLsmXF/oLmsXR (Table S3). In addition, sequences coding the His_6_ tag and a TEV cleavage site were fused to the N-terminus of the Lsm1 protein. The seven coding sequences were assembled in a stepwise manner in the pBS3021 backbone giving plasmid pBS5031.

The Lsm1-7 complex from *S. cerevisiae* was expressed using pBS5031 plasmid in *E. coli* BL21(DE3) Gold (Stratagene) in 1 L of autoinducible medium at 37°C supplemented with kanamycin at 100 µg/mL. Cells were harvested by centrifugation and resuspended in 35 mL of buffer C (20 mM Tris-HCl pH 7.5, 500 mM NaCl, 25 mM imidazole, 5 mM 2-mercaptoethanol). Cell lysis was performed by sonication. The complex was purified on a NiNTA column (Qiagen). Eluted Lsm1-7 proteins were incubated overnight with TEV protease in dialysis buffer (20 mM Tris-HCl pH 7.5, 150 mM NaCl, 0.5 mM EDTA, 5 mM 2-mercaptoethanol). The His-tagged TEV protease and uncleaved complex were removed by incubation with NiNTA resin. The untagged Lsm1-7 complex was present in the flow-through and further purified by ion exchange chromatography at pH 7.5 (HiTrap Q FF, GE Healthcare) followed by size-exclusion chromatography on Superdex 200 16/60 column (GE Healthcare) in buffer D (20 mM Tris-HCl pH 7.5, 150 mM NaCl, 5 mM 2-mercaptoethanol).

### Pull-down assays

Pull-down experiments were performed by mixing 52.5 µg of His_6_ tagged Pat1 [435-796] with 2 fold molar excess amounts of untagged Lsm1-7 complex. Binding buffer (20 mM Tris-HCl pH 7.5, 100 mM NaCl, 50 mM imidazole, 10% Glycerol) was added to a final volume of 60 µL. The reaction mixtures were incubated on ice for 2 hours. 10 µL was withdrawn to constitute the input fraction. The rest was incubated with 500 µg of HisPur Ni-NTA Magnetic Beads (Thermo Scientific) in a final volume of 200 µL at 4°C for 2 hours. Beads were washed three times with 500 µL of binding buffer. Bound proteins were eluted with 250 mM imidazole. Samples were resolved on SDS-PAGE and visualized by Coomassie blue staining.

### Crystallization and structure determination

Needle crystals were initially obtained for the ScPat1C domain in a wide range of conditions from two commercial kits (MB Class I and Procomplex from Qiagen) and some conditions were optimized to obtain diffracting crystals. All the crystals were cryo-protected by transfer into their crystallization condition with progressively higher ethylene glycol concentration up to 30% and then flash-cooled in liquid nitrogen. All datasets were collected on beam-line Proxima-1 (Synchrotron SOLEIL, Saint-Aubin, France).

A first 4 Å resolution dataset (crystal form I) was collected at Se-edge from a crystal of Se-Met labeled protein obtained by mixing 1 µL of ScPat1C at 17 mg/mL in buffer A with an equal volume of crystallization solution (4% PEG 8,000; 0.1 M Tris-HCl pH 7.5). This dataset was processed with the MOSFLM and SCALA programs ([52], [53], Table 1). This crystal belongs to space group P2_1_ with two or three molecules in the asymmetric unit, corresponding to a solvent content of 63% or 44%, respectively. The ScPat1C structure was solved by Se-SAD using this dataset. The SHELXD program was used to find an initial set of 12 Se sites in the 48-5 Å resolution range [54]. Refinement of the Se sites and phasing were carried out with the SOLVE program [55]. Solvent flattening and NCS averaging were performed with the program RESOLVE [56]. The best experimental electron density maps were obtained for a solvent content of 65%, indicating that the asymmetric unit only contains two copies of the ScPat1C domain. Although of poor quality due to the limited resolution, these experimental maps allowed the building of several poly-Ala alpha-helices.

**Table 1:** Data collection and refinement statistics.

	Crystal form I (Se-Met)	Crystal form II	Crystal form III
**Data collection**			
Space group	P2_1_	P2_1_2_1_2_1_	P2_1_
Cell dimensions			
*a*, *b*, *c* (Å)	112.6, 37.6, 116.8	36.3, 173.15, 175	55.2, 88.6, 68.1
α, β, γ (°)	90, 99, 90	90, 90, 90	90, 96.1, 90
Resolution (Å)	48–4.0 (4.22–4.0)	30–2.3 (2.41–2.3)	50–2.15 (2.27–2.15)
Total number of reflections	92,290	189,218	142,169
Total number of unique reflections	8,604	49,604	35,407
*R*_sym_ a	0.113 (0.53)	0.054 (0.56)	0.081 (0.57)
*I*/σ*I*	11.8 (5.3)	15.7 (2.4)	11.3 (2.1)
Completeness (%)	99.6 (100)	99.6 (98.2)	98.9 (95.8)
Redundancy	10.7	3.8	4
**Refinement**			
Resolution (Å)		30–2.3	50–2.15
*R*_work_/*R*_free_ (%)^b^		24/30.4	21/26.5
R.m.s. deviations			
Bond lengths (Å)		0.012	0.008
Bond angles (°)		1.45	1.064
**PDB code**		4OJJ	4OGP

a R_sym_ = ∑_h_∑_i_|I_hi_ - <I_h_>|/∑_h_∑_i_I_hi_, were I_hi_ is the *i* ^th^ observation of the reflection h, while <I_h_> is the mean intensity of reflection h.

b R_factor_ = ∑ ||F_o_| - |F_c_||/|F_o_|. R_free_ was calculated with a small fraction (5%) of randomly selected reflections.

In parallel, two additional datasets (crystal forms II and III) could be collected from crystals of native protein (10 mg/mL in buffer A) grown in 20% PEG 4,000; 50 mM sodium citrate; 5% isopropanol and 15% PEG 6,000; 7.5% MPD; 0.1 M MES pH 6.5, respectively. These datasets were processed with the XDS package [57]. Crystal form II belongs to space group P2_1_2_1_2_1_ with three ScPat1C copies in the asymmetric unit and yielded a 2.3 Å resolution dataset. Crystal form III diffracted to 2.15 Å resolution and belongs to space group P2_1_ with 2 ScPat1C molecules in the asymmetric unit. Statistics on data collection and refinement are gathered within Table 1. The dataset collected from crystal form II was then used for molecular replacement trials with the MOLREP program [58] to locate 3 copies of the poly-Ala model built in the 4 Å resolution experimental map (crystal form I). Successive cycles of building/refinement with the COOT [59] and PHENIX.REFINE [60] programs yielded a final model with R and R_free_ values of 24% and 30.4%, respectively. The final model obtained from crystal form II is incomplete due to the lack of electron density for certain protein regions, most probably due to their intrinsic flexibility. The final model contains residues Gly474 to Tyr787 from monomer A, Glu480 to Ile483, Phe523 to Gln694, Asp700 to Leu 795 from monomer B and Lys476 to Lys796 (as well as the three first residues from the His-tag) from monomer C. In addition, one ethylene glycol molecule from the cryo-protection solution, one magnesium ion, one chloride ion and 231 water molecules were modeled in the 2Fo-Fc electron density map.

The dataset collected from crystal form III was also used to refine the structure of ScPat1C domain. Two copies of the Pat1C model were positioned by molecular replacement with MOLREP and refined using the PHENIX.REFINE program to reach final R and R_free_ values of 21% and 26.5%, respectively (Table 1). The final model contains residues Gly474 to Ile644 and Leu654 to Lys796 to from monomer A, Gly473 to Ser648 and Leu654 to Lys796 from monomer B as well as 2 MES buffer molecules, 3 ethylene glycol molecules from the cryo-protection solution and 141 water molecules.

## Results

### Delineation of ScPat1 C domain and structure determination

As previous studies have emphasized on the role of ScPat1 C domain (region 422-796) in translation repression [21], in reduction of 48S translation initiation intermediates accumulation [26] and in localization to P-bodies [21], we decided to determine its crystal structure to obtain information on its organization at the atomic level. Analysis of the sequence of the ScPat1 C domain using bioinformatics tools (*i.e*. secondary structure prediction, HCA “Hydrophobic Cluster Analysis” tool [61]) and sequence conservation among yeast species revealed that the region 422-472 is poorly conserved and is particularly enriched in Ser, Ala, Asn and basic residues (Lys/Arg), which are characteristics of unstructured regions (data not shown). On the opposite, the region encompassing residues 473 to 796 displays significant sequence identity within yeast species and is predicted to be well folded. We therefore cloned the ScPat1C (473-796) domain, over-expressed it in *E. coli* and purified it in milligram amounts to homogeneity. The SeMet labeled and unlabeled proteins crystallized in three different space groups (Table 1). Given the much lower resolution of the dataset collected from crystal form I SeMet protein crystals, this dataset was only used to obtain experimental electron density maps by the Single wavelength Anomalous Diffusion method (SAD) using the selenium anomalous signal but not for structure refinement. Only structures refined to higher resolution (crystal forms II and III) will be discussed in this manuscript.

Three and two copies of the ScPat1C molecule are present in the asymmetric unit of crystal forms II and III, respectively. All these structures can be superimposed onto each other with rmsd values ranging from 0.3 Å to 1.2 Å over 260-310 Cα atoms. The relatively high rmsd values observed between some molecules result from flexibility of both N- and C-terminal extremities of this domain (Fig.S1A). This flexibility is also supported by the observation that residues 484-524 from one protomer as well as residues 788-796 (corresponding to a β-sheet, see below) from another one are ill-defined in the electron density maps and hence absent from the final model. In parallel, two independent structures of the ScPat1C domain bound to Lsm1-7 complex [39] or Lsm2-Lsm3 subcomplex [40] were recently determined at 3.7 Å and 3.15 Å resolution, respectively (rmsd values ranging from 0.9 Å to 1.6 Å with our structures of ScPat1C domain). In these structures, the C-terminal extremity of the ScPat1C domain exhibited higher flexibility than the remaining parts of the domain. This is illustrated either by the absence of the C-terminal β-hairpin and of residues 743-764 (corresponding to the end of helix α14 and the beginning of helix α15) in the structure of the Pat1-Lsm1-7 complex or by the high B-values of the residues 644-794 in the structure of the Pat1-Lsm2-3 subcomplex compared to the other residues from this domain. Finally, the comparison of nine independent crystal structures has also highlighted flexibility of the N-terminal end of human Pat1b C-terminal domain [36]. This conserved characteristic suggests that flexible regions located at both ends of ScPat1C domain might be of functional importance.

The crystal packing radically differs between crystal forms II and III. In crystal form III, the two Pat1 molecules of the asymmetric unit exhibit a very intricate head-to-tail organization with an interacting surface area of 1,635 Å^2^. As this value is in the range expected for interfaces within homodimers [62], this suggested that this ScPat1C domain could also exist as a homodimer in solution. To investigate this possibility, we have analysed the quaternary structure of ScPat1C in solution using both size exclusion chromatography coupled online to a triple detection array (MALLS, Fig.S1B) and small angle X-ray Scattering (SAXS; Fig.S1C). MALLS measurements on a protein at 2 mg/mL yielded a molecular mass of 39.3 kDa, indicating that ScPat1C is monomeric in solution (the theoretical molecular weight for the monomer is 38.6 kDa). Furthermore, the SAXS experimental curves measured at various ScPat1C concentrations (5.4 and 11.2 mg/mL, *i.e*. concentrations closer to those used in the crystallization experiments) clearly correspond to the curve calculated from the coordinates of the ScPat1C monomer but not from those of the crystal dimer (Fig.S1C). This SAXS analysis indicates that: first, the large contact area observed in crystal form III is created by the crystal-packing and does not reflect a biologically relevant oligomeric state; second, ScPat1C adopts in solution a structure similar to that observed in the crystals.

### *Saccharomyces cerevisiae* Pat1C is a member of the ARM repeat superfamily

**Figure 1:**
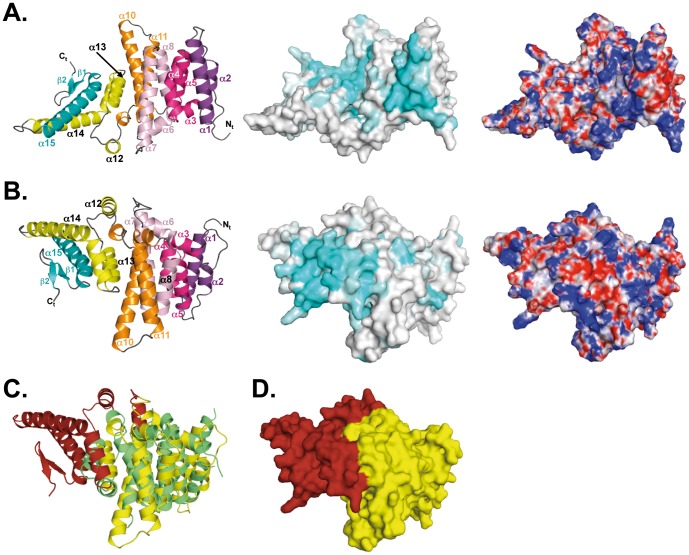
Structure of *S. cerevisiae* Pat1 C domain. A and B. Left panels: ribbon representation of ScPat1C crystal structure. The ARM repeats are depicted using different colors. Middle panel: Mapping of the sequence conservation at the surface of the ScPat1C domain. Coloring is from grey (low conservation) to cyan (highly conserved). The conservation score was calculated using the CONSURF server [67] and using an alignment made from the sequences of 30 Pat1 fungal orthologues. Right panel: Mapping of the electrostatic potential at the surface of the ScPat1C domain. Positively (10 k_B_T/e^-^) and negatively (−10 k_B_T/e^-^) charged regions are colored in blue and red, respectively. The electrostatic potential was calculated using PBEQ Solver server [68]. Orientation in B differs from A by a 180° rotation along the horizontal axis. C. Ribbon representation of the superimposition between human Pat1C domain (green; [36]) and ScPat1C domain. The core conserved from yeast to human is colored in yellow. The fungi specific C-terminal extension from ScPat1C is colored in red. D. Surface representation of ScPat1 domain. Same color code as panel C.

The structure of the C-terminal half of ScPat1 is formed by 15 α-helices followed by two C-terminal β strands. The α-helix packing of the protein adopts an α-α superhelix fold reminiscent of the ARM repeat superfamily. ScPat1C is organized as an array of 5 repeats of 3 helices stacked against each other to form a solenoid structure (Fig.1A–B). Within each repeat, the second and third helices are packed in an anti-parallel manner and the shorter first helix is perpendicular to the two other helices [63]. Within the superhelix, the second and third helices from a repeat are stacked against the second and third helices from the neighboring repeats, respectively. The first helices from each repeat are all exposed to the same face of the solenoid. In the ScPat1C domain, the first helix from repeat 1 is absent and the length of the ARM repeats ranges in length from around 40 to 70 amino acids. The fifth and final motif is capped at its C-terminal end by helix α15 followed by a two-stranded anti-parallel β-hairpin, which folds back onto helices α14 and α15. These two latter helices are long (25 to 30 residues long) and protrude from the core of the protein.

**Figure 2:**
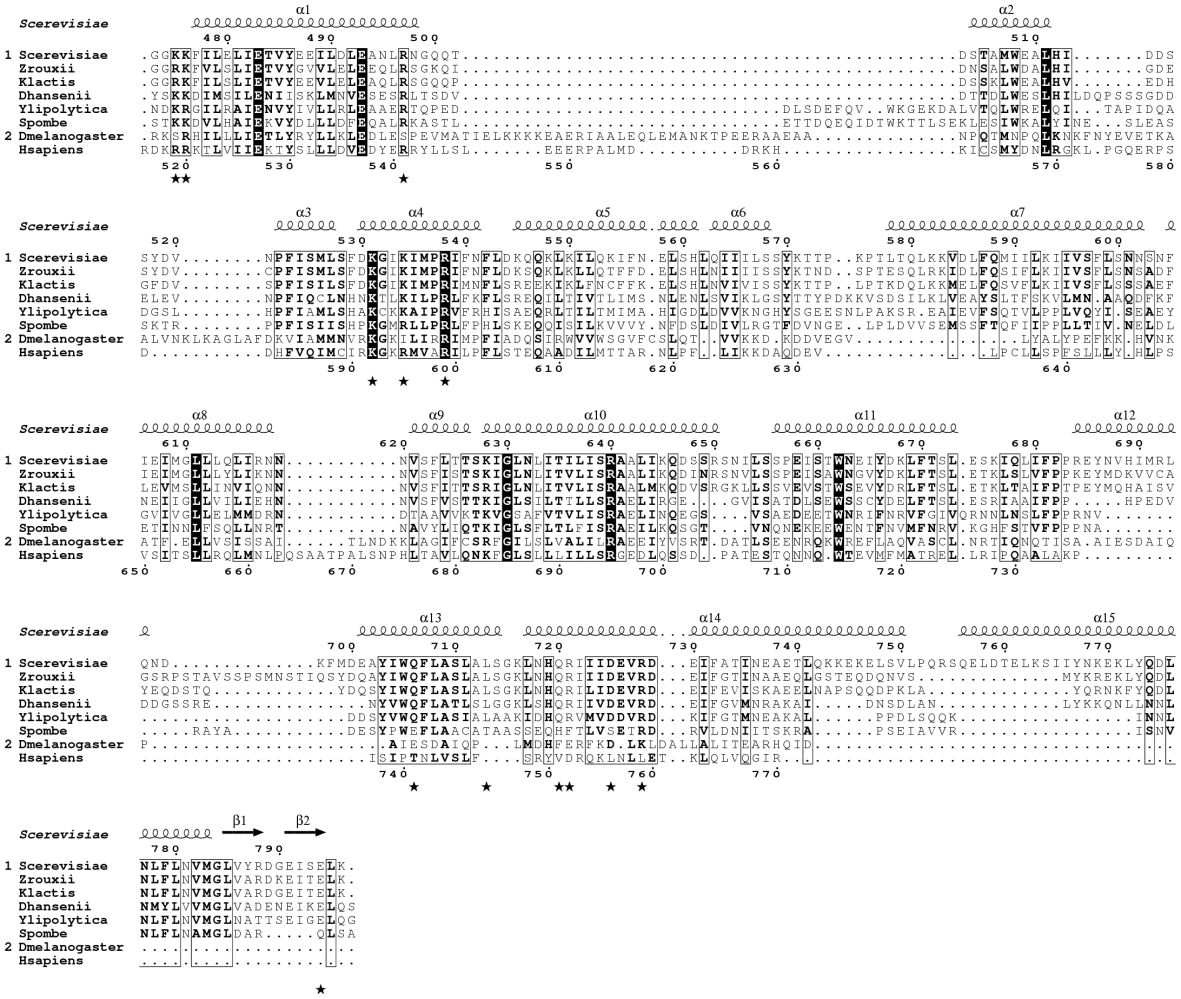
Sequence alignment of Pat1 orthologues. Alignment was performed using ClustalW [69]. Strictly conserved residues are in white on a black background. Partially conserved amino acids are boxed. Secondary structure elements assigned from the ScPat1C structure are indicated above the alignment. Black stars below the sequences indicate residues mutated in this study. This figure was generated using the ESPript server [70].

The ScPat1C domain adopts the same fold as the corresponding domain from human Pat1b (rmsd value of 3.4–3.8 Å over 200 Cα atoms and 20% sequence identity, Fig.1C, [36]). The highest structural similarity is found in the region encompassing residues 476 to 680 from yeast (520–732 from human Pat1b). The C-terminal part of this domain (680–796 in yeast and 733–765 in human) is much more divergent and in particular, *S. cerevisiae* protein exhibits a significantly longer C-terminal extension with two long α-helices (α14, α15) and a two stranded β-hairpin (Fig.1A-B & D). This C-terminal extension, which also encompasses helix α12, is specific for fungi and varies in length among yeast species (Fig.2).

Mapping of the sequence conservation at the surface of the ScPat1C reveals the presence of two highly conserved regions (Fig.1A-B). The first region is conserved both in fungal and in metazoan proteins and has very recently been shown to be directly involved in the interaction with the Lsm1-7 complex [39], [40]. It is mainly formed by residues located within helices α1 (Lys475, Lys476, Glu483, Glu493 and Arg497) and α4 (Lys531, Lys534 and Arg538) and displays a positively charged electrostatic potential (Fig.1A; Fig.2). The other conserved region is located at the opposite side of the domain and corresponds to the fungi specific region (Fig.1B; Fig.2). This region is formed by the C-terminal end of ScPat1C (helices α13, α14, α15 and strands β1 and β2) and is electrostatically neutral.

### The conserved positively charged N-terminal region from ScPat1C involved in the interaction with Lsm1-7 complex, is important for yeast growth at 37°C

**Figure 3:**
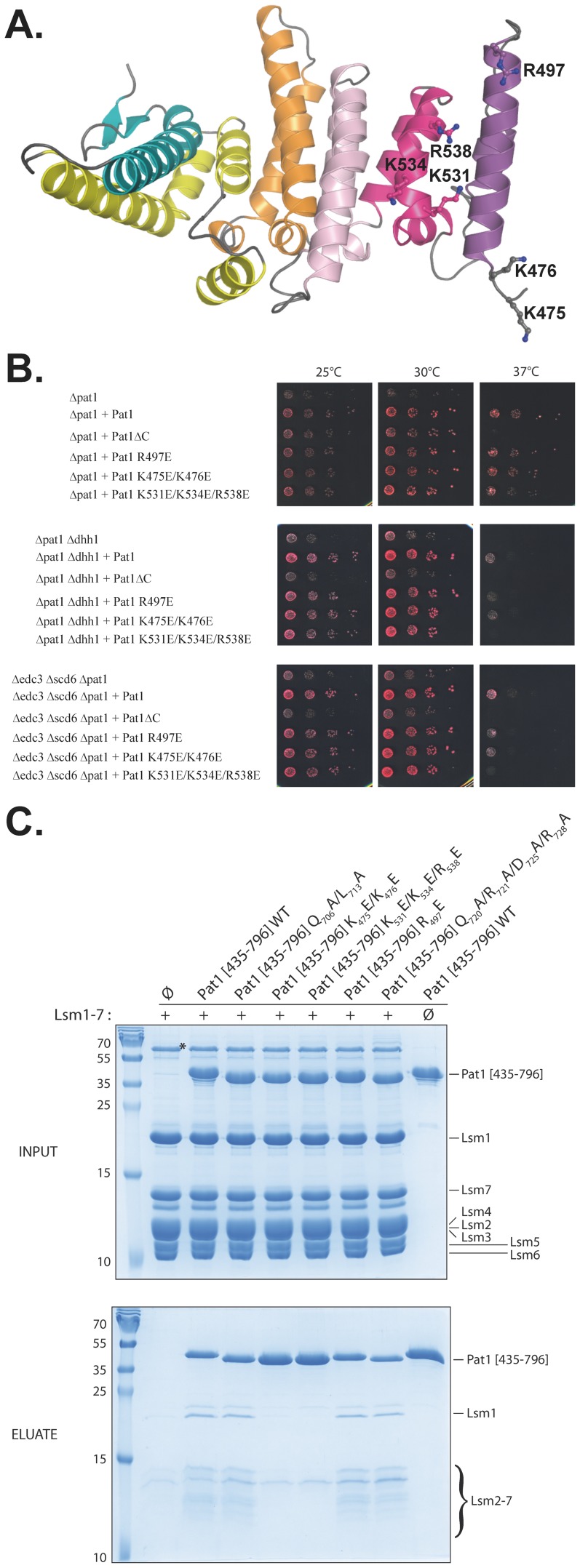
The conserved residues at the N-terminal residues are involved in interaction with Lsm1-7. A. ScPat1C structure with the residues mutated in this study shown as ball and sticks. Color scheme identical to Fig.1A. B. Growth analysis of *PAT1* mutants. Serial dilutions of the different strains transformed with vector, a plasmid encoding wild type ScPat1, or the indicated mutant thereof, were deposited on plates and incubated at the indicated temperatures. C. Pull-down experiment of untagged Lsm1-7 complex with His_6_ ScPat1C wild type or mutants. Input (top) and eluted (bottom) samples were analyzed by 22% SDS-PAGE and Coomassie Blue staining. The asterisk denotes a contaminant protein that co-purifies with the Lsm1-7 complex but which is not retained on the HisPur™ Ni-NTA Magnetic Beads used for these pull-down experiments. Molecular weights (kDa) of the markers are indicated on the left of the gels.

To test for the importance of the conserved region located at the N-terminal extremity of ScPat1 domain, we have used complementation of Pat1 mutants (Fig.3). Since Pat1 is known to collaborate with other factors to induce mRNA decay and repress translation, we first constructed strains carrying deletion of *PAT1* alone or in combination with *DHH1*, or with *EDC3* and *SCD6* deletions. Consistent with previous observation, deletion of *PAT1* resulted in slow growth phenotype at 25°C or 30°C and thermosensitivity at 37°C (Fig.3B; [15], [16], [19]). Exacerbated phenotypes, supporting partly redundant functions, were observed in both the *edc3Δ/scd6Δ/pat1Δ* and *dhh1Δ/pat1Δ* strains. This is consistent with previous observations and with the participation of these factors in translation repression and/or mRNA decay [64]. Full-length Pat1 protein fully complements for the deletion of the *PAT1* gene in all these strains while a Pat1 fragment lacking the crystallized ScPat1C domain (Pat1 [1–472] or Pat1ΔC) does not, indicating that this C-terminal domain is functionally important (Fig.3B). Based on this observation, we next mutated residues located within the conserved N-terminal region from ScPat1C by substituting lysine and arginine residues from helices α1 and α4 by glutamic acid (Fig.3A). This yielded three charge-inversion mutants (K475E/K476E, R497E and K531E/K534E/R538E) whose ability to complement for *PAT1* deletion was tested in the three different genetic backgrounds. While the R497E and K475E/K476E mutants behave mostly as the wild-type ScPat1 protein, the K531E/K534E/R538E mutant exhibits a slightly reduced complementation in the *pat1Δ* strain (detected as smaller colonies), and does not complement for *PAT1* deletion in *dhh1Δ* and *edc3Δ/scd6Δ* strains (Fig.3B). Furthermore, as no complementation defects are detectable at 25°C or 30°C for these three mutants, we conclude that these mutants are functional and well folded. This was confirmed by normal accumulation of these different point mutant proteins in yeast cells (Fig.S2). Altogether, these complementation analyses indicate that the positively charged conserved region and in particular K531, K534 and R538 residues, found at the N-terminal region of ScPat1C play an important functional role, that is more easily detected in the absence of the Dhh1 helicase or of the Edc3 and Scd6 proteins.

These functionally important conserved residues are located within the ScPat1C region interacting with the Lsm1-7 complex [39], [40]. As the charge inversion mutants that we have tested in our phenotypic analyses were not generated previously (only substitutions of some of these residues by Ala were performed [39], [40]), we have investigated whether the complementation defects observed for the ScPat1C mutants could result from the inability of our mutants to interact with Lsm1-7 complex. We have purified the Lsm1-7 complex as well as various ScPat1C mutants following over-expression in *E. coli* and performed pull-down assays. As shown in Fig.3C, the untagged Lsm1-7 complex was specifically retained on NiNTA beads when incubated with the wild-type His_6_ tagged ScPat1 fragment encompassing residues 435-796 (*i.e.* slightly longer than ScPat1C). Similarly, the R497E mutant as well as two other mutants targeting residues located within the opposite C-terminal extension present in Pat1 fungal proteins (Q706A/L713A and Q720A/R721A/D725A/R728A) interact to the same extent as the wild-type ScPat1 domain. Interestingly, the K475E/K476E and K531E/K534E/R538E ScPat1 mutants failed to interact with the Lsm1-7 complex, confirming that the conserved positively charged residues (K475, K476, K531, K534 and R538) located within this N-terminal region from ScPat1C are directly involved in the interaction with the Lsm1-7 complex. Furthermore, in the case of the K531E/K534E/R538E ScPat1 mutant, the disruption of Lsm1-7-Pat1C interaction suggests that this interaction is crucial for yeast growth at 37°C in the absence of Scd6 and Edc3 proteins (Fig.3B). Although we did not detect interaction between K475E/K476E ScPat1C mutant and the Lsm1-7 complex *in vitro*, this mutant complements for the deletion of *PAT1* gene in the different background tested. This could indicate that this later mutant retains some residual binding activity towards the Lsm1-7 complex *in vivo* and this would be sufficient to support yeast growth at 37°C (see discussion).

### The yeast specific C-terminal extension from ScPat1C is involved in Edc3 binding

**Figure 4:**
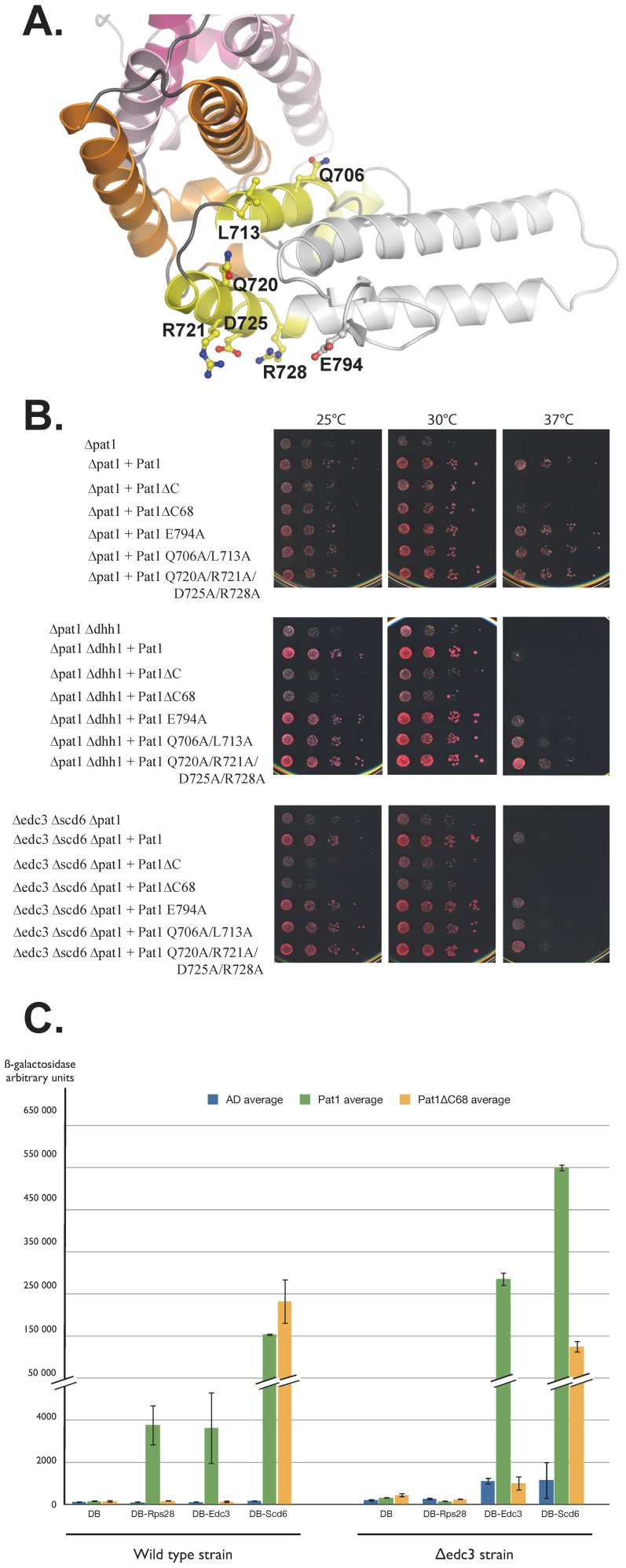
The yeast specific residues located at the C-terminus of ScPat1C are functionally important. A. ScPat1C structure with the residues mutated in this study shown as ball and sticks. Color scheme identical to Fig.1A. The region absent in the ScPat1ΔC68 construct is colored in grey. B. Growth analysis of *PAT1* mutants. Serial dilutions of the different strains transformed with vector, a plasmid encoding wild type ScPat1, or the indicated mutant thereof, were deposited on plates and incubated at the indicated temperatures. C. Monitoring ScPat1 interaction with Edc3, Rps28 or Scd6 using the two-hybrid assay. The wild-type or *edc3Δ* strain was transformed with the indicated pairs of vectors and interaction between the factors encoded by these two plasmids was scored by assaying β-galactosidase activity.

We next investigated whether the second conserved region, *i.e.* the fungi specific C-terminal extension is important for Pat1 biological function (Fig.4). We used the same yeast strains to assess the ability of ScPat1 lacking residues 730 to 796 from this C-terminal extension (ScPat1ΔC68) to complement for the deletion of *PAT1* gene (Fig.4B). This truncated protein was unable to fully restore growth at 37°C upon expression in all mutant strains with the strongest phenotypes again observed in the *pat1Δ*/*scd6Δ/edc3Δ* and *pat1Δ*/*dhh1Δ* strains (Fig.4B). A *pat1*Δ strain carrying a deletion of the ScPat1 C-terminal extension grows only slightly better than a strain with Pat1ΔC and both deletions have similar effects in the *pat1Δ*/*dhh1Δ* and *pat1Δ*/*scd6Δ/edc3Δ* backgrounds. Hence, our data support that this C-terminal extension plays a functional role, whose importance is better detected in the more sensitive double mutant strains.

We next investigated the importance of well-conserved residues within this region by substituting them by Ala to yield three different mutants: Q706A/L713A, Q720A/R721A/D725A/R728A and E794A (Fig.4A). All these mutants were expressed at similar levels as the wild-type protein in yeast (Fig.S2) and fully complemented for PAT1 deletion in the three backgrounds tested indicating that they are not affected in their function. Surprisingly, the *pat1Δ*/*scd6Δ/edc3Δ* and *pat1Δ*/*dhh1Δ* strains expressing the Q706A/L713A and Q720A/R721A/D725A/R728A mutants grew faster at 37°C than the same strains expressing full-length wild-type Pat1 (Fig.4B).

To go deeper into the role of this Pat1 fungi specific C-terminal extension, we tested the ability of full-length ScPat1 and ScPat1ΔC68 to interact with Rps28, Edc3 and Scd6 in a two-hybrid assay (Fig.4C). In a wild type strain background, we could detect the Pat1-Edc3 interaction that was previously reported [21] as well as positive signals for Pat1-Rps28 and Pat1-Scd6. The association with Rps28 and Scd6 were not previously observed using the two-hybrid assay but independent evidences support our observations. Indeed, an interaction between Pat1 and Scd6 was observed using purified factors [26]. Similarly, Rps28 is known to interact with Edc3 which itself binds Pat1 [30]. Interestingly, neither Edc3 nor Rps28 interact with the ScPat1ΔC68 protein while Scd6 interacts as efficiently as with the full-length Pat1 protein (Fig.4C). The latter observation indicates that ScPat1ΔC68 is well folded and functional. In the *Δedc3* host strain, Rps28 was unable to interact with wild type ScPat1 revealing thereby that Edc3 is necessary to bridge the two factors. We observed that the Pat1-Edc3 interaction signal was much stronger (more than 60 fold) in the *Δedc3* strain compared to wild type. This indicates that endogenous Edc3 competes with Edc3 fused to the DNA binding domain for interacting with Pat1. Even with the very high signal observed in this context, deletion of the C-terminal region of Pat1 totally abolished the interaction with Edc3 (Fig.4C). The Scd6-Pat1 interaction signal was only slightly enhanced in the Δ*edc3* strain (3-fold) and remained strongly positive with the ScPat1ΔC68 in this context. Thus, Scd6 interacts with Pat1 through an interface that remains to be identified but that differs at least in part from the one mediating Edc3 binding, as it does not require the fungal C-terminal extension of Pat1. Consistently, Edc3 competed only weakly with the Scd6-Pat1 interaction.

In summary, these data demonstrate that the C-terminal extension specific to fungal Pat1 proteins plays an important functional role, probably through its ability to mediate interaction with protein partners including Edc3.

## Discussion

The Pat1 protein is involved in different aspects of mRNA decapping activation. Indeed, Pat1 represses translation initiation with its Dhh1 and Scd6 partners [22], [26], [43] but also activates the Dcp1-Dcp2 enzyme together with Edc3 and, in yeast, Edc1 and Edc2 [26], [31]. Pat1 also interacts with the Lsm1-7 complex, which binds to the 3′ end of oligoadenylated mRNAs, thereby connecting 3′ to 5′ and 5′ to 3′ mRNA decay pathways [15], [16], [20], [34], [65]. Hence, Pat1 is considered as a central platform protein for decapping. Our present study further reinforces this idea. Indeed, the crystal structure of the *S. cerevisiae* Pat1C shows that this domain structurally belongs to the ARM repeat superfamily, which mostly encompasses proteins involved in protein-protein interaction. This is consistent with already published data that have identified many protein partners for this domain (Lsm1-7, Xrn1, Dcp1, Dcp2, Edc3 in yeast, human and/or drosophila as well as EDC4 in human; [15], [20], [26], [34], [36]). Furthermore, sequence conservation mapping at the surface of ScPat1C domain reveals the presence of conserved residues clustered at both extremities of this domain and involved in protein recruitment by Pat1.

The first conserved region is located at the N-terminal part of the ScPat1C domain and the corresponding region from HPat was previously shown to be involved in the recruitment of the LSM1-7 complex, DCP2 and EDC4 proteins by co-immunoprecipitation experiments performed in *D. melanogaster* cells [36]. Recently, two independent crystal structures of ScPat1C-Lsm1-7 and ScPat1C-Lsm2-3 complexes clearly showed that this region (and in particular residues K475, K476, K531, K534 and R538) is involved in the interaction with Lsm3 within the Lsm1-7 complex [39], [40]. Furthermore, the mutation of some of these residues resulted in mRNA stabilization by altering mRNA decapping and 3′→5′ decay *in vivo* [40]. We have observed that this N-terminal conserved region from Pat1C is indeed of functional importance as illustrated by the thermosensitive phenotype observed for *pat1Δ*/*scd6Δ/edc3Δ* and *pat1Δ*/*dhh1Δ* yeast strains expressing the Pat1 K531E/K534E/R538E mutant (Fig.3B). We further confirmed that this ScPat1 mutant, which was not generated in the other studies, fails to interact directly with the Lsm1-7 complex using *in vitro* pull-down assays (Fig.3C). Contrary to the K531E/K534E/R538E mutant, the K475E/K476E mutant does not interact with Lsm1-7 complex *in vitro* but fully complements for the deletion of ScPat1 *in vivo* (Fig.3B-C). This most probably results from the location of the K475 and K476 residues at the periphery of the ScPat1C-Lsm1-7 interface while the K531, K534 and R538 residues are located at the center of this interaction [39], [40]. Indeed, one can speculate that the interaction between the Lsm1-7 complex and ScPat1C K475E/K476E mutant domain is too weak to resist the conditions of the *in vitro* pull-down assay performed with purified truncated Pat1 proteins while, *in vivo*, a residual interaction probably strengthened by the bridging activity of other decapping factors is likely to take place with this full-length ScPat1 mutant and to be sufficient for function. Altogether, these different studies rationalize the functional importance of these conserved Pat1 residues and indicate that the physical interaction between Pat1 and the Lsm1-7 complex is crucial for yeast cell growth at 37°C in the absence of Dhh1 or Edc3 and Scd6 proteins. This further strongly suggests that the growth defects that we observe for the K531E/K534E/R538E mutant is most likely the result of the inability of this mutant to interact with Lsm1-7 complex and then to activate mRNA decapping and 3′→5′ decay *in vivo*.

Opposite to the ScPat1C region involved in the interaction with Lsm1-7 complex, there is a fungi-specific C-terminal extension (encompassing residues 730–796 from ScPat1), which is important for growth at 25°C, 30°C and 37°C in the absence of the *DHH1* gene or of both *EDC3* and *SCD6* genes (Fig.4B). Expression of the ScPat1ΔC68 protein in a *Δpat1* background also results in a slight growth defect at 37°C. Furthermore, we have observed using yeast two-hybrid that ScPat1ΔC68 no longer interacts with Edc3 and Rps28 (Fig.4C). The interaction with Rps28 requires Edc3, which itself interacts with Rps28 through a conserved linear motif [30]. However, the ScPat1ΔC68 protein still binds to Scd6, indicating that this truncated version of the protein is still present and folded in yeast. A previous report indicated that residues 697–763 from ScPat1 are required for recruitment of Lsm1-GFP to P-bodies and for the interaction of Pat1 with Edc3 but not Lsm1 [21]. As our structural information indicate that this internal deletion affects both the conserved and non-conserved regions of ScPat1, it was not possible to conclude about the role of the yeast specific region (residues 729 to 796) from these data. Our results indicate that it is required for Edc3 binding but it remains to be determined whether the Pat1-Edc3 interaction is direct or bridged by other decapping factors. Indeed, ScPat1C domain was shown to interact physically with Dcp2, which in turn should interact with Edc3 Lsm domain through several short helical leucine-rich motifs (HLM) as shown in *S. pombe* [24], [26]. Furthermore, no interaction between Edc3 and Pat1 proteins was detected by co-immunoprecipitation in *D. melanogaster* cells while the association described for human proteins was proposed to be indirect [20], [34]. Hence, the involvement of the ScPat1 C-terminal and fungal specific extension in the interaction with Edc3 is of particular interest. Other species-specific variations in the interaction network of decapping factors have been observed. For example, while the interaction between Dcp1 and Dcp2 is direct in yeast, it is strengthened by EDC4/Hedls in human [32], [33]. Similarly, the Edc3 and Scd6 Lsm domains interact with Dcp2 through short helical leucine-rich motifs (HLM) in *S. pombe* while in metazoan, the HLM motif involved in EDC3 binding is present in DCP1 [24]. It has been proposed that phylogenetic variations in this protein interaction network arise through the appearance of Short Linear Interaction Motifs (SLiMs), which are intrinsically disordered when unbound [66]. While the appearance and evolution of such motifs are easy to envisage, our observation that yeast Pat1 proteins contain a C-terminal extension that is structured and required for Edc3 binding, indicates that evolution can also promote the formation of more complex and structured protein appendages that facilitate protein association.

In conclusion, the crystal structure of the ScPat1 C-terminal domain has revealed two functionally important regions localized at opposite extremities of the domain. One region is enriched in positively charged residues and is important for yeast growth at 37°C. It is involved in direct binding to the Lsm1-7 complex that is required for decapping activation. The second region is required for the interaction of ScPat1 with Edc3 and its precise role remains to be elucidated.

## Supporting Information

Figure S1 A. Superimposition of the five ScPat1C structures (each ScPat1C structure is shown with a different color). The rmsd values range from from 0.3 Å to 1.2 Å over 260-310 Cα atoms. B. Size-exclusion chromatogram of ScPat1C is shown. For clarity, only the refractive index (RI, red, left y axis) for the eluted sample and the molecular mass calculated from light scattering (right y axis, black, logarithmic scale) are shown. C. Comparison between the experimental curve (open circles) obtained for ScPat1C and the curve (red line) calculated using the program CRYSOL from the crystal structure of the Pat1 monomer to which the missing C-ter His_6_ tag was added [7]. The excellent agreement (χ = 1.56) between these curves proves unambiguously that ScPat1C is a monomer in solution at low concentration below a few mg/mL and that the structure of the protein in solution is comparable to the crystal structure. For comparison the curve calculated from the coordinates of the crystal dimer (dashed gray line) is also shown.
(TIF)

Figure S2 Western blot analysis of protein levels for full-length and point mutant derivatives of ScPat1. Lsm1 was used as a loading control.
(TIF)

Table S1 Yeast strains used in this study.
(DOCX)

Table S2 Yeast shuttling plasmids used in this study.
(DOCX)

Table S3 Oligonucleotides used in this study.
(DOCX)

File S1 Supplementary methods and associated references.
(DOCX)
