# Supplementary figures and images for: The C-Terminal Domain from S. cerevisiae Pat1 Displays Two Conserved Regions Involved in Decapping Factor Recruitment
Source: PLoS One. 2014 May 15;9(5):e96828. doi: 10.1371/journal.pone.0096828 (PMC4022514; doi:10.1371/journal.pone.0096828)

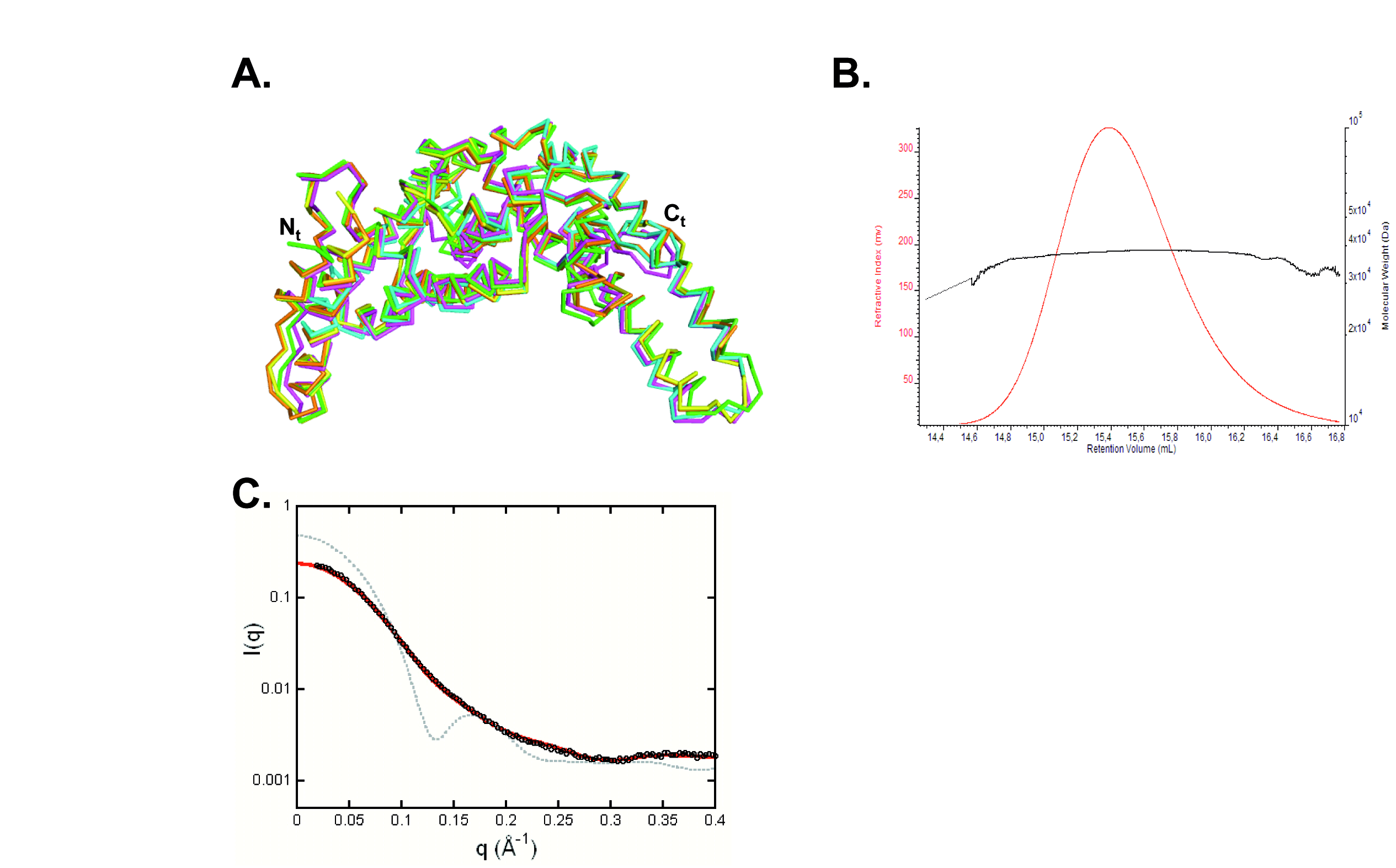

Supplement: Figure S1 — A. Superimposition of the five ScPat1C structures (each ScPat1C structure is shown with a different color). The rmsd values range from from 0.3 Å to 1.2 Å over 260-310 Cα atoms. B. Size-exclusion chromatogram of ScPat1C is shown. For clarity, only the refractive index (RI, red, left y axis) for the eluted sample and the molecular mass calculated from light scattering (right y axis, black, logarithmic scale) are shown. C. Comparison between the experimental curve (open circles) obtained for ScPat1C and the curve (red line) calculated using the program CRYSOL from the crystal structure of the Pat1 monomer to which the missing C-ter His6 tag was added [7]. The excellent agreement (χ = 1.56) between these curves proves unambiguously that ScPat1C is a monomer in solution at low concentration below a few mg/mL and that the structure of the protein in solution is comparable to the crystal structure. For comparison the curve calculated from the coordinates of the crystal dimer (dashed gray line) is also shown. (TIF) [file pone.0096828.s001.tif]

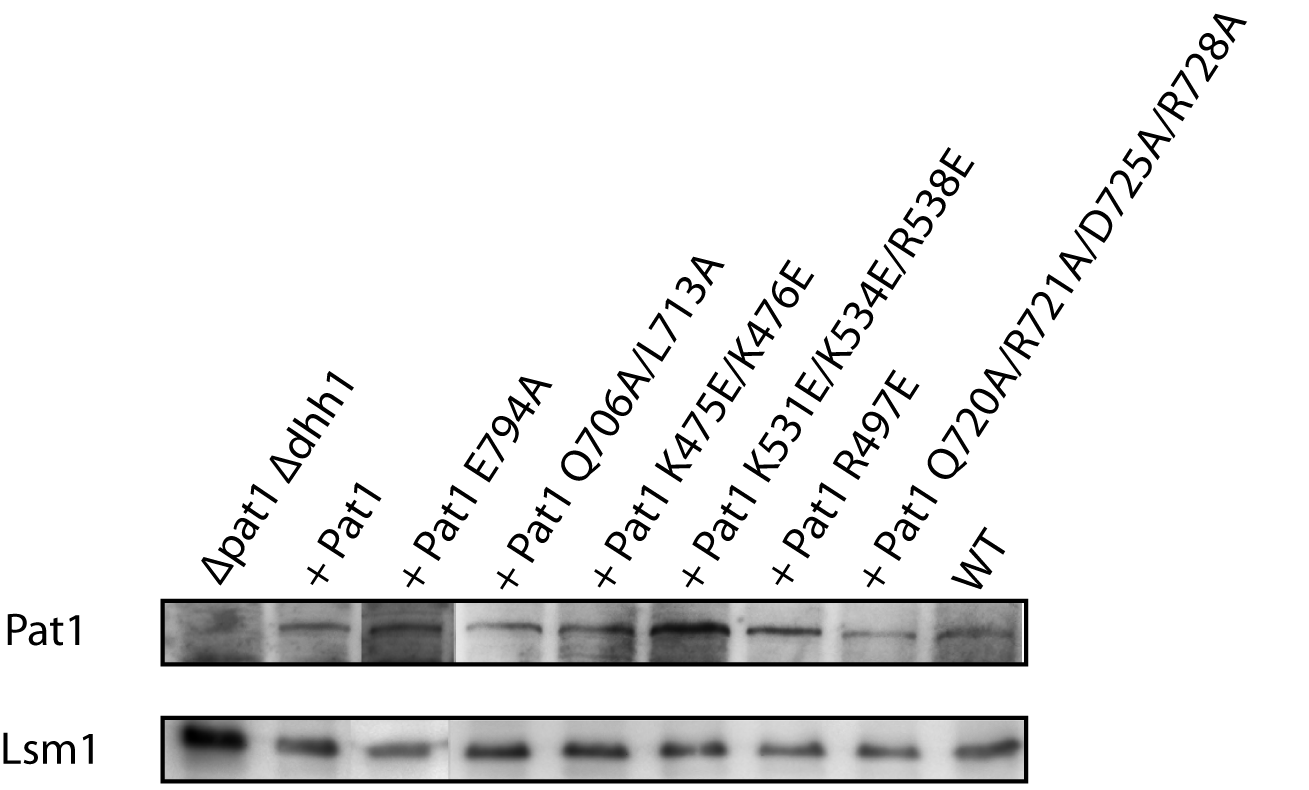

Supplement: Figure S2 — Western blot analysis of protein levels for full-length and point mutant derivatives of ScPat1. Lsm1 was used as a loading control. (TIF) [file pone.0096828.s002.tif]
