# Supplementary material for: The C-Terminal Domain from S. cerevisiae Pat1 Displays Two Conserved Regions Involved in Decapping Factor Recruitment
Source: PLoS One. 2014 May 15;9(5):e96828. doi: 10.1371/journal.pone.0096828 (PMC4022514; doi:10.1371/journal.pone.0096828)
Supplement: Table S1 — Yeast strains used in this study. (DOCX) [file pone.0096828.s003.docx]

***Table S1: Yeast strains used in this study***

| **Strain** | **Genotype** | **Reference** |
| --- | --- | --- |
| BMA64 | MATα; ura3-1; ∆trp1; ade2-1; leu2-3,112; his3-11,15 | [4] |
| MAV203 | MATα; leu2-3,112; trp1-901; his3∆200; ade2-101; gal4∆; gal80∆; SPAL10::URA3; GAL1::lacZ; HIS3UAS GAL1::HIS3@LYS2; can1^R^; cyh2^R^ | Invitrogen |
| BSY1133 | MATa; ura3-1; trp1-1; ade2-1; leu2-3,112; his3-11,15; ∆pat1::HIS3 | Gift from Michèle Minet |
| BSY2475 | MATα leu2-3,112, trp1-901, his3-∆200, ade2-101, gal4∆, gal80∆, SPAL10::URA3, GAL1::lacZ, HIS3UAS GAL1::HIS3@LYS2, can1^R^, cyh2^R^, ∆edc3::NAT | [5] |
| BSY 2601 | MATa; ura3-1 or ura3-52; trp1-1 or ∆trp1; ade2-1; leu2-3,112; his3-11,15; ∆pat1::HIS3; ∆scd6::Kan^R^; ∆edc3::Nat^R^ | This work |
| YFW168 | MATa; ura3-1; ∆trp1; ade2-1; leu2-3;112; his3-11,15; ∆pat1::HIS3; ∆dhh1::Kan^R^ | Gift from Michèle Minet |
