# Supplementary material for: The C-Terminal Domain from S. cerevisiae Pat1 Displays Two Conserved Regions Involved in Decapping Factor Recruitment
Source: PLoS One. 2014 May 15;9(5):e96828. doi: 10.1371/journal.pone.0096828 (PMC4022514; doi:10.1371/journal.pone.0096828)
Supplement: Table S2 — Yeast shuttling plasmids used in this study. (DOCX) [file pone.0096828.s004.docx]

***Table S2: Yeast shuttling plasmids used in this study***

| **Plasmid** | **Description** | **Reference or oligonucleotides used for construction** |
| --- | --- | --- |
| pRS414 | Shuttling vector with TRP1 marker | [6] |
| pDEST22 | 2 hybrid vector with NLS and GAL4 activation domain (GAL4AD), TRP1 marker | Invitrogen |
| pDEST32 | 2 hybrid vector with DNA binding domain, LEU2 marker | Invitrogen |
| pBS2366 | pDEST32, carrying a sequence encoding yeast Rps28a | [5] |
| pBS2368 | pDEST32 carrying a sequence encoding Edc3 | Gift from N. Cougot |
| pBS2374 | Two-hybrid plasmid expressing Pat1 fused to the Gal4AD in the pDEST22 backbone. | Gift from N. Cougot |
| pBS3910 | pDEST32 carrying a sequence encoding Scd6 | OBS4236  OBS4237 |
| pBS4357 | Wild type PAT1 gene in pRS414 backbone | OBS4954  OBS4955 |
| pBS4421 | Pat1∆C68 derivative of pBS4357 | OBS5012  OBS5013 |
| pBS4423 | Pat1-E794A derivative of pBS4357 | OBS5020  OBS5021 |
| pBS4425 | Derivative of pBS4357 lacking residues 473-796 | OBS5108/OBS5109 |
| pBS4436 | Pat1-Q706A/L713A derivative of pBS4357 | OBS5157/OBS5158 |
| pBS4437 | Pat1-K475E/K476E derivative of pBS4357 | OBS5149  OBS5150 |
| pBS4438 | Pat1-K531E/K534E/R538E derivative of pBS4357 | OBS5153  OBS5154 |
| pBS4439 | Pat1-R497E derivative of pBS4357 | OBS5151  OBS5152 |
| pBS4440 | Pat1- Q720A/R721A/D725A/R728A derivative of pBS4357 | OBS5159 OBS5160 |
| pBS4907 | Derivative of pBS2374 expressing pat1∆C68 ; obtained by QuickChange mutagenesis | OBS5012  OBS5013 |
