# Supplementary material for: The C-Terminal Domain from S. cerevisiae Pat1 Displays Two Conserved Regions Involved in Decapping Factor Recruitment
Source: PLoS One. 2014 May 15;9(5):e96828. doi: 10.1371/journal.pone.0096828 (PMC4022514; doi:10.1371/journal.pone.0096828)
Supplement: Table S3 — Oligonucleotides used in this study. (DOCX) [file pone.0096828.s005.docx]

***Table S3: Oligonucleotides used in this study***

| **Oligonucleotide** | **Sequence** |
| --- | --- |
| oMG18 | AGACTAGCTAGCGGGGGCAAAAAATTCATTCTTGAG |
| oMG118 | TTTTTGCGGCCGCTTACTTTAGTTCTGATATTTCACCATCG |
| oMG119 | GGGAAGCTAGCATGCACCATCACCATCACCATTCTTCAGGGTCCTCCTCTAC |
| oMG180 | TTTTTGCGGCCGCTTAATGGTGATGGTGATGGTGCTTTAGTTCTGATATTTC |
| OBS4236 | GGGGACAAGTTTGTACAAAAAAGCAGGCTTCATGTCGCAGTACATCGGTAAAACT |
| OBS4237 | GGGGACCACTTTGTACAAGAAAGCTGGGTCTTAAAATTCAACGTTGGAAGGAGG |
| OBS4954 | GCGCCTCGAGGCCCCGATTGCAGCCTGTTATTG |
| OBS4955 | GCGCGGATCCAGTTCAACCTTATTCTCAATC |
| OBS5012 | GAAGTACGTTAAGATTTTTCCTGTAATTTTAATGTACTTACGC |
| OBS5013 | GAAAAATCTTAACGTACTTCATCAATAATGATTCTCTGG |
| OBS5020 | AATATCAGCGCTAAAGTAAGATTTTTCCTG |
| OBS5021 | TTACTTTAGCGCTGATATTTCACCATCGCG |
| OBS5108 | AAATTTGAACAAATCTTAAGATTTTTCCTGTAATTTTAATGTACTTACGC |
| OBS5109 | AAATTACAGGAAAAATCTTAAGATTTGTTCAAATTTGTGGCACCATTACCG |
| OBS5149 | ATCTGGGGGCGAAGAATTCATTCTTGAG |
| OBS5150 | AAGAATGAATTCTTCGCCCCCAGATTTGTTC |
| OBS5151 | GAAGCTAACTTGGAGAATGGCCAGCAAAC |
| OBS5152 | CTGGCCATTCTCCAAGTTAGCTTCCAAGTC |
| OBS5153 | GCTATCATTTGATGAAGGTATCGAGATTATGCCTGAAATTTTTAATTTCTTGG |
| OBS5154 | TTAAAAATTTCAGGCATAATCTCGATACCTTCATCAAATGATAGCATCG |
| OBS5157 | ATACATTTGGGCGTTCCTAGCTAGTTTAGCAGCAAGTGGAAAGCTAAACC |
| OBS5158 | GCTTTCCACTTGCTGCTAAACTAGCTAGGAACGCCCAAATGTATGCTTC |
| OBS5159 | GCTAAACCACGCGGCAATCATTATTGCTGAAGTAGCTGATGAAATCTTTGCC |
| OBS5160 | GATTTCATCAGCTACTTCAGCAATAATGATTGCCGCGTGGTTTAGCTTTCC |
| OBS6352 | GGCTCTAGATAAGGAGGATATATATGGAGACACCTTTGGATTT |
| OBS6353 | GCCGGCGCGCCTCATATCTCCACTGCGCCATCGT |
| OBS6354 | GGCGGATCCTAAGGAGGATATATATGCACCATCACCATCACCATTCTGCAAATAGCAAGGAC |
| OBS6355 | GCCGAGCTCTTAGTACATGTCAGATTTAT |
| oLsm2F | GGCGGCGCGCCTAAGGAGGATATATATGCTTTTCTTCTCCTTTTT |
| oLsm2R | GCCGGATCCTTATTTTCTTTCAGTCATTA |
| oLsm4F | GGCGAGCTCTAAGGAGGATATATATGCTACCTTTATATCTTTT |
| oLsm4R | GCCGCTAGCTTAGTTGGAGTTAATTTGCT |
| oLsm7F | GGGCCTAGCTAAGGAGGATATATATGCATCAGCAACACTCCCA |
| oLsm7R | GCCGGCCGGCCCTATTTTTGCATATATAGTA |
| oLsm5F | GGCGGCCGGCCTAAGGAGGATATATATGAGTCTACCGGAGATTTT |
| oLsm5R | GCCTTAATTAATTACAACGCCTCCGTAGGGG |
| oLsm6F | GGCTTAATTAATAAGGAGGATATATATGTCCGGAAAAGCTTCTAC |
| oLsm6R | GCCGGTACCCTATATTTTTTGTTCACTGA |
