# Supplementary material for: The C-Terminal Domain from S. cerevisiae Pat1 Displays Two Conserved Regions Involved in Decapping Factor Recruitment
Source: PLoS One. 2014 May 15;9(5):e96828. doi: 10.1371/journal.pone.0096828 (PMC4022514; doi:10.1371/journal.pone.0096828)
Supplement: File S1 — Supplementary methods and associated references. (DOCX) [file pone.0096828.s006.docx]

**Supplementary files**

**The C-terminal domain from *S. cerevisiae* Pat1 displays two conserved regions involved in decapping factor recruitment.**

Zaineb Fourati^1,2,*^, Olga Kolesnikova^3,*^, Régis Back^1^, Jenny Keller^2^, Clément Charenton^1^, Valerio Taverniti^3^, Claudine Gaudon-Plesse^3^, Noureddine Lazar^2^, Dominique Durand^2^, Herman van Tilbeurgh^2^, Bertrand Séraphin^3^, Marc Graille^1,2^

^1^ Laboratoire de Biochimie, Centre National de Recherche Scientifique (CNRS) UMR 7654, Ecole Polytechnique, F-91128 Palaiseau Cedex, France.

^2^ Institut de Biochimie et Biophysique Moléculaire et Cellulaire (IBBMC), Centre National de Recherche Scientifique (CNRS) UMR 8619, Bat. 430, Université Paris Sud, F-91405 Orsay Cedex, France.

^3^ Equipe Labellisée La Ligue, Institut de Génétique et de Biologie Moléculaire et Cellulaire (IGBMC), Centre National de Recherche Scientifique (CNRS) UMR 7104/Institut National de Santé et de Recherche Médicale (INSERM) U964/Université de Strasbourg, 67404 Illkirch, France

**Supplementary methods.**

*Multi-Angle Laser Light Scattering (MALLS)*

Sample injection, chromatography and detection were carried out using a Triple Detector Array (TDA305) system coupled in line to a GPCmax chromatographic system (Viscotek). A volume of 100 µL of Pat1 (2 mg/mL) was injected at a flow rate of 0.5mL/min on a Superdex 200 HR 10/30 column (GE Healthcare) equilibrated with 20 mM Tris-HCl pH 7.5, 200 mM NaCl, 5 mM β-mercaptoethanol. Elution was followed by a UV-Visible spectrophotometer, a differential refractometer, a 7° Low Angle Light Scattering detector, a 90° Right Angle Light Scattering detector and a differential pressure viscometer. The data were collected and processed with the program OmniSEC 4.7 (Viscotek). The *dn/dc* value for BSA (0.185) was used in the calculation.

*Small Angle X-ray Scattering (SAXS)*

SAXS experiments were carried out using the Nanostar instrument (Brüker, Karlsruhe, Germany) with X-rays generated by a rotating anode (wavelength λ=1.54 Å). The useful scattering vector range was 0.012 < q < 0.40 Å^-1^, where q=4πsinθ/λ and 2θ is the scattering angle. SAXS data were normalized to the intensity of the transmitted beam, put to an absolute scale using the scattering by water and background subtracted using the program package PRIMUS [1]. Data were collected in a buffer composed of 20 mM Tris-HCl pH 7.5, 200 mM NaCl at two protein concentrations (11.2 and 5.4 mg/mL). Due to a slight effect of attractive interactions detectable in the low-q range, data were extrapolated to zero concentration. The extrapolated curve and the one collected at 11.2 mg/mL were merged to obtain a scattering curve free from inter-particle interactions and of good statistic in the high-q range.

*Western blot analyses*

For Western blot analysis 1ml of cell cultures were grown in SC medium w/o tryptophan till OD_600_ ≥2. Total yeast extract was prepared as described previously [2]. For detection a rabbit serum containing polyclonal antibodies generated against a fragment of Pat1 was used (1:1000; kind gift from F. Wyers, [3]). For a loading control, the endogenous Lsm1 protein was detected using polyclonal anti-Lsm1 antibody (1:1000; kind gift from F. Wyers).

**References.**

1. Konarev PV, Volkov VV, Sokolova AV, Koch MHJ, Svergun DI (2003) PRIMUS - a Windows-PC based system for small-angle scattering data analysis. J Appl Cryst 36: 1277-1282.

2. Kushnirov VV (2000) Rapid and reliable protein extraction from yeast. Yeast 16: 857-860.

3. Wyers F, Minet M, Dufour ME, Vo LT, Lacroute F (2000) Deletion of the PAT1 gene affects translation initiation and suppresses a PAB1 gene deletion in yeast. Mol Cell Biol 20: 3538-3549.

4. Baudin-Baillieu A, Guillemet E, Cullin C, Lacroute F (1997) Construction of a yeast strain deleted for the TRP1 promoter and coding region that enhances the efficiency of the polymerase chain reaction-disruption method. Yeast 13: 353-356.

5. Kolesnikova O, Back R, Graille M, Seraphin B (2013) Identification of the Rps28 binding motif from yeast Edc3 involved in the autoregulatory feedback loop controlling RPS28B mRNA decay. Nucleic Acids Res 41: 9514-9523.

6. Sikorski RS, Hieter P (1989) A system of shuttle vectors and yeast host strains designed for efficient manipulation of DNA in Saccharomyces cerevisiae. Genetics 122: 19-27.

7. Svergun DI, Barberato C, Koch MHJ (1995) CRYSOL - a Program to Evaluate X-ray Solution Scattering of Biological Macromolecules from Atomic Coordinates. J Appl Cryst 28: 768-773.
